# Investigation of the polyamine biosynthetic and transport capability of *Streptococcus agalactiae*: the non-essential PotABCD transporter

**DOI:** 10.1099/mic.0.001124

**Published:** 2021-12-15

**Authors:** Sarah Khazaal, Rim Al Safadi, Dani Osman, Aurélia Hiron, Philippe Gilot

**Affiliations:** ^1^​ ISP, Bactéries et Risque Materno-Foetal, Université de Tours, INRAE, 37032 Tours, France; ^2^​ Azm Center for Research in Biotechnology and its Applications, LBA3B, EDST, Lebanese University, Tripoli, 1300, Lebanon

**Keywords:** ABC transporter, oxidative stress, polyamines

## Abstract

Polyamines constitute a group of organic polycations positively charged at physiological pH. They are involved in a large variety of biological processes, including the protection against physiological stress. In this study, we show that the genome of *Streptococcus agalactiae *, a commensal bacterium of the intestine and the vagina and one of the most common agents responsible of neonate infections, does not encode proteins homologous to the specific enzymes involved in the known polyamine synthetic pathways. This lack of biosynthetic capability was verified experimentally by TLC analysis of the intracellular content of *S. agalactiae* grown in the absence of polyamines. However, similar analyses showed that the polyamines spermidine, spermine and putrescine can be imported from the growth media into the bacteria. We found that all strains of *S. agalactiae* possess the genes encoding the polyamine ABC transporter PotABCD. We demonstrated that these genes form an operon with *folK*, a gene involved in folate biosynthesis, *murB*, a gene involved in peptidoglycan biosynthesis, and with *clc*, a gene encoding a Cl^−^/H^+^ antiporter involved in resistance to acid stress in *Escherichia coli *. Transcription of the *potABCD* operon is induced by peroxide-induced oxidative stress but not by acidic stress. Spermidine and spermine were found to be inducers of *potABCD* transcription at pH 7.4 whereas putrescine induces this expression only during peroxide-induced oxidative stress. Using a deletion mutant of *potABCD*, we were nevertheless unable to associate phenotypic traits to the PotABCD transporter, probably due to the existence of one or more as yet identified transporters with a redundant action.

## Introduction

Polyamines, small aliphatic hydrocarbon molecules with a quaternary nitrogen chemical group, have a net positive charge at physiological pH. They are associated with a large range of biological functions such as efficient DNA replication, transcription, translation, stress resistance, cell proliferation and differentiation [1, 2]. Together with Mg^2+^ and Ca^2+^, polyamines constitute the major polycations in cells. They are able to bind to intracellular polyanions such as nucleic acids and ATP to modulate their functions. Putrescine (1,4-diaminobutane), spermidine [*N*-(3-aminopropyl)butane-1,4-diamine], spermine [*N*,*N*′-*bis* (3-aminopropyl)butane-1,4-diamine] and cadaverine (1,5-diaminopentane) are the most widely distributed cellular polyamines and are essential for normal multiplication and cellular growth of most prokaryotic and eukaryotic cells [2]. Polyamines appear to play a crucial role in the pathogenesis and virulence of important human bacterial pathogens. Several studies, in species such as *Escherichia coli* and *Streptococcus pneumoniae,* involved polyamines in the protection of bacterial cells from the toxic effects of reactive oxygen, by their function of radical scavengers [3, 4]. In addition, polyamines are key mediators in the resistance to acidic stress in several bacterial species. For example, in *E. coli* and *Salmonella enterica *, they induce the expression of amino acid decarboxylases, which are directly involved in the response of these bacteria to acidic stress, and thus facilitate their survival *in vivo* [5, 6]. The pleiotropic effects of polyamines on nucleic acid stability, transcription and translation also play an important role in the physiological adaptation of *S. pneumoniae* during temperature stress [4]. The physiological functions of polyamines cannot take place without a highly regulated level of intracellular polyamines, which is based on the coordination of the processes of the polyamine uptake, synthesis and degradation [2]. Most prokaryotes have *de novo* biosynthesis pathways in which polyamines are generated via enzymatic modification of amino acid precursors [7]. In addition, almost all bacteria possess polyamine transport systems to satisfy their requirements from the environment [8].

**Fig. 1.**
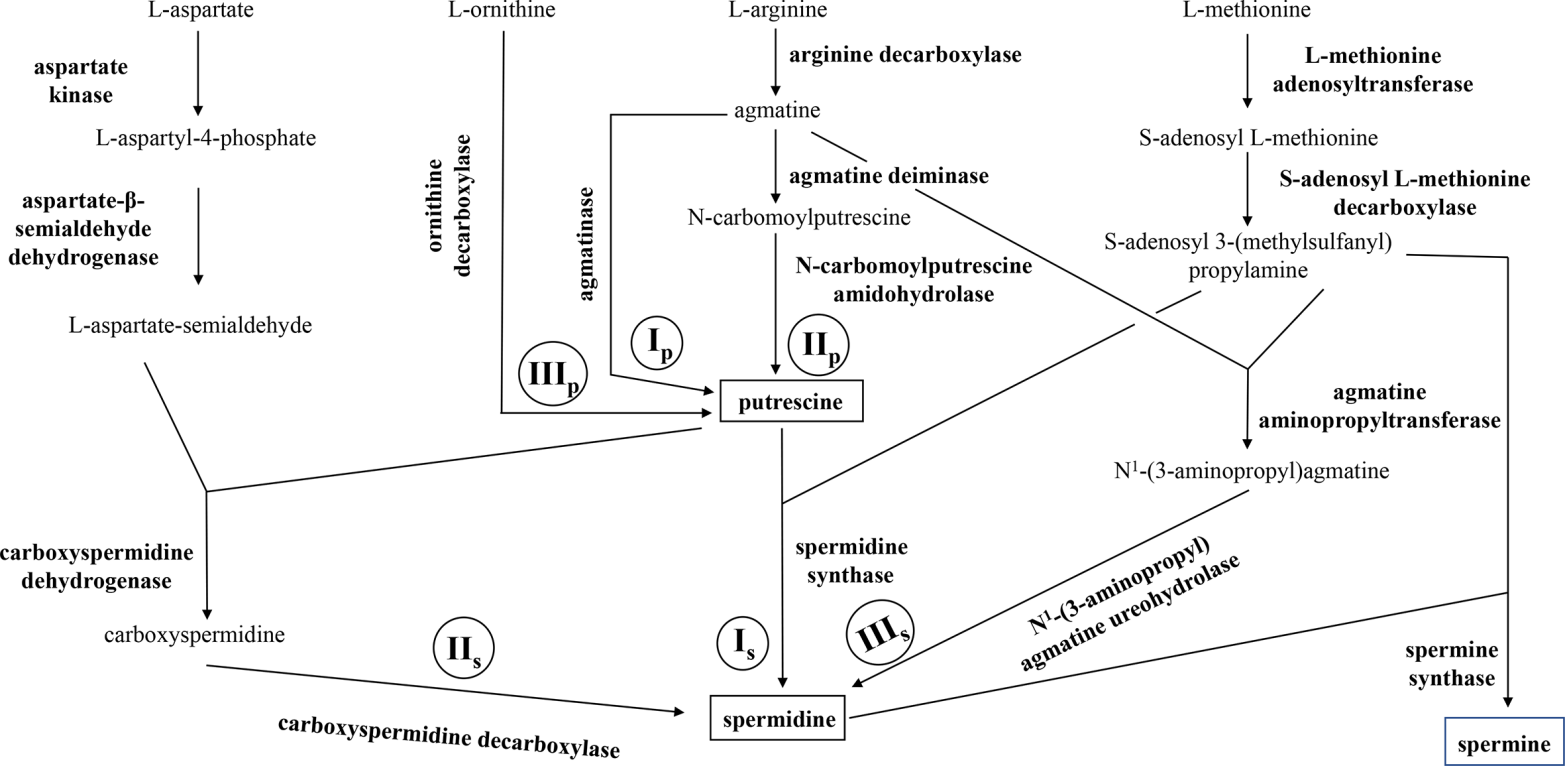
Known *de novo* polyamine biosynthetic pathways in microorganisms. Three pathways for the biosynthesis of putrescine (Ip, IIp, IIIp), two for the biosynthesis of spermidine (Is, IIs) and one for the biosynthesis of spermine are described in microorganisms.

Three pathways of putrescine biosynthesis (Ip, IIp and IIIp; Fig. 1) and three pathways of spermidine biosynthesis (Is, IIs and IIIs; Fig. 1) have been described in bacteria [9–16]. In *Saccharomyces cerevisiae,* spermine can be synthesized by the addition of a propylamine group to spermidine, a reaction catalysed by spermine synthase (Fig. 1) [17]. However, although the presence of spermine is attested to in several bacterial species, no specific bacterial spermine synthase has yet been discovered [18]. Polyamines are not biosynthesized by all bacterial species. For example, in the genus *Streptococcus *, while polyamine biosynthesis pathways (IIp, Is and IIs) are present in all strains of *S. pneumoniae *, they are lacking in the majority of the strains of *Streptococus suis* and *Streptococcus mitis,* which must acquire polyamines from the environment [19].

Almost all bacteria can also import extracellular polyamines by a polyamine ATP-binding cassette (ABC) transporter, encoded by an operon of four genes [4, 20, 21]. Some bacteria, such as *E. coli *, possess two polyamine ABC transporters, PotABCD and PotFGHI, having a higher affinity for either spermidine or putrescine, respectively [22, 23]. Only a single *potABCD* operon is detected in many other bacteria, such as *Staphylococcus aureus *, *S. pneumoniae *, *S. suis* or *Streptococcus agalactiae* [19, 24–27]. PotA and PotG are membrane-associated cytosolic ATPases. PotB with PotC or PotH with PotI constitute transmembrane channels for polyamine transport. Located in the periplasm or anchored to the cytoplasmic membrane, PotD and PotF are substrate-binding proteins that trap extracellular polyamines [22, 23]. The binding of polyamine to the substrate-binding proteins results in a conformational change of the membrane spanning proteins of the transporter, which leads to ATP hydrolysis and polyamine uptake. The presence of excess polyamines in the environment can function as a feedback regulator on polyamine transport. It was shown in *E. coli* that a high concentration of spermidine can inhibit the polyamine transport system, by the inhibition of ATPase activity, through the interaction of spermidine with a domain of PotA [28]. In addition, PotD is also able to inhibit the transcription of the *potABCD* operon of *E. coli* [8].

*S. agalactiae,* also called Group B *Streptococcus *, was first distinguished from other streptococci by Rebecca Lancefield in 1930, after it was isolated from milk, and was detected as a primordial cause of mastitis in cows [29]. It is a Gram-positive, β-haemolytic bacterium, which frequently and asymptomatically colonizes the gastrointestinal and/or urogenital tract of humans [30]. *S. agalactiae* maternal carriage was identified as a high risk factor for the development of neonatal disease and preterm birth [31]. In neonates, *S. agalactiae* is one of the leading causes of invasive infections, such as pneumonia, septicaemia and meningitis [32]. It has also emerged as an increasingly common cause of invasive diseases in immunocompromised and elderly adults [33]. In addition to its ability to colonize the gastrointestinal and uro-genital tracts, *S. agalactiae* also colonizes the throat or the oral and nasopharyngeal mucosa. Furthermore, it is able to infect the amniotic and cerebrospinal fluids, the blood and the mammary gland [34–37]. Moreover, *S. agalactiae* can contaminate foodstuffs, and it has been isolated from pastries and seafood products [38]. Such ability to survive in many different environments indicates its large capability for adaptation. As polyamines are involved in the resistance mechanism of several bacterial species to environmental stress, they could be involved in the substantial ability for adaptation of *S. agalactiae *. However, the physiological role of polyamines and the phenotypic traits given by the PotABCD transporter have not yet been studied in *S. agalactiae *.

In the genome of all *Streptococcus* species*,* a gene called *murB*, which encodes an enzyme implicated in peptidoglycan biosynthesis, is localized upstream of the *potABCD* operon [19]. We previously identified the transcriptional promoter of *murB* in the *folK–murB* intergenic region of *S. agalactiae *. We also localized another transcriptional promoter in the upstream *folK* gene and we showed that genes involved in folate biosynthesis are co-transcribed with *murB*. This co-transcription could be necessary to synchronize two processes of cell wall synthesis, as it was postulated that a folic acid-mediated reaction might be involved in cell wall synthesis [39, 40]. In some *S. agalactiae* strains, the mobile element IS*1548* is inserted in the *folK–murB* intergenic region. The presence of this insertion sequence prevents the co-transcription of *murB* with genes of the folate pathway. However, as IS*1548* brings an additional promoter able to initiate *murB* transcription, the insertion of IS*1548* results in a minor negative modulation of the expression of *murB* [40]. In *S. pneumoniae* and *S. suis,* it was demonstrated that *murB* is co-transcribed with *potABCD*, which suggests also a relationship between polyamine transport and peptidoglycan biosynthesis in streptococci [19, 41]. Polyamines were described to be components of the peptidoglycan. In *S. pneumoniae *, putrescine can substitute for choline, which is involved in peptidoglycan synthesis and hydrolysis [41]. In *S. suis *, peptidoglycan synthesis and separation of daughter cells during cell division cannot be completed without the presence of polyamines [19]. In Gram-negative bacteria, spermidine, putrescine and cadaverine are also considered as constituents of the peptidoglycan, since they play a significant role in maintaining cell shape and integrity of the cell surface structure [42, 43]. In *S. agalactiae *, the *clc* gene is located downstream of the *potABCD* genes. The *murB*, *potABCD* and *clc* genes are all transcribed in the same direction. The *clc* gene is predicted to code a Cl^−^/H^+^ antiporter [27]. Cl^−^/H^+^ antiporters found in *E. coli* promote proton expulsion and were described to be highly induced under acid stress [44]. The presence of *clc* downstream of *potABCD* is noteworthy since, as discussed above, polyamines are involved in the resistance of some bacteria to acidic stress.

In this study, we first analysed the genome of *S. agalactiae* strains available at the National Center for Biotechnology Information (NCBI) database to look for the presence of genes encoding enzymes involved in known spermine, spermidine and putrescine biosynthesis pathways. Similarly, the prevalence of the *potABCD* operon in *S. agalactiae* strains was evaluated by blast analysis of completely sequenced genomes. The polyamine biosynthetic and transport capability of *S. agalactiae* was tested by TLC analyses of the intracellular polyamine content of bacteria grown in the absence or in the presence of polyamines. We then examined the transcription pattern of the *potABCD* region and analysed the expression of the *pot* operon in response to polyamines and various stress conditions. The influence of polyamines on the growth and on the survival of *S. agalactiae* was finally determined.

## Methods

### Plasmids, bacterial strains and growth conditions

The plasmids and bacterial strains used in this study are listed in Table 1. *E. coli* strains were cultured in liquid LB medium (MP Biomedicals; Cat. no. 3002022) or on LB-agar plates (1.5 % agar). Liquid cultures of *E. coli* were agitated at 200 r.p.m. at 37 °C. *S. agalactiae* strains were routinely grown on 5 % horse blood trypticase soy (TSH) agar plates (1.5 % agar) (bioMérieux; Cat no. 43061) or on Todd Hewitt (TH) agar plates (BD Bacto; Cat. no. 249240). Liquid cultures of *S. agalactiae* were performed at 37 °C without agitation in TH broth (BD Bacto; Cat. no. 249240) adjusted to pH 7.4 with HCl. For the maintenance of pG +host1 derivatives, *E. coli* and *S. agalactiae* strains were grown with erythromycin (150 µg ml^−1^ for *E. coli* or 10 µg ml^−1^ for *S. agalactiae *).

**Table 1.** Bacterial strains and plasmids used in this study

Strain or plasmid	Genotype or description	Source or reference
* E. coli* strain		
XL1-blue	*endA1 gyrA96* (Nal^R^) *thi-1 recA1 relA1 lac glnV44 hsdR17*(r_K-_ m_K+)_ F' [ ::Tn*10* (Tet^R^) *proAB* ^+^ *lacI^q^ ZΔM15*]	Stratagene
* S. agalactiae* strains		
A909	Isolated from a septic human neonate in 1934 (ST 7, CC 7)	[40]
A909∆*potABCD*	Isogenic *potApotBpotCpotD* (*sak_1196, sak_1195, sak_1194 and sak_1193*) deletion mutant of A909	This study
Plamids		
pG+host1^TS^	Replication-thermosensitive shuttle plasmid, Ery^R^	[48]

### Liquid chemically defined media for growth of *S. agalactiae*

The liquid chemically defined medium used to grow *S. agalactiae* (CDM) contains 8.3 g l^–1^ Dulbecco’s modified Eagle medium base (catalogue no. D5030; Sigma-Aldrich), 1× BME vitamins, 74 µM adenine, 89.2 µM uracil, 65.7 µM xanthine, 66.2 µM guanine, 1123.5 µM d,l-alanine, 757 µM l-asparagine, 1127 µM l-aspartic acid, 684.5 µM l-glutamine, 1019.5 µM l-glutamic acid, 868.6 µM l-proline, 734.9 µM l-tryptophan, 4125.4 µM l-cysteine, 12 µM lipoic acid, 1 µM pyruvate, 17.4 µM ZnSO_4_.7H_2_O, 10.5 µM CoCl_2_.H_2_O, 0.4 µM CuSO_4_.5H_2_O and 55 mM d-glucose. This medium was finally adjusted to pH 7.4 with HCl. For some experiments, CDM was buffered at pH 7.4 with 100 mM HEPES (Fisher Bioreagents; cat. no. BP310-100), to pH 5.5 with 100 mM MES (ACROS Organics; code no. 172591000) or pH 4.0 by the addition of 33.5 mM sodium citrate dihydrate and 66.5 mM citric acid. Final adjustments of the pH were made with citric acid or HCl. Spermidine (ACROS Organics; Cat. No. AC132740050), putrescine (ACROS Organics; Cat. No. AC132750050) or spermine (ACROS Organics; Cat. No. AC112120250) were also added for some experiments.

### Measurement of bacterial growth

For measuring bacterial growth in chemically defined medium, *S. agalactiae* strains were first cultured in TH broth at pH 7.4 (without agitation) to the stationary phase of growth. These cultures were centrifuged and washed in non-buffered CDM at pH 7.4. They were then suspended to an OD_600 nm_ of 0.005 in the same medium and grown overnight at 37 °C, without agitation. These last cultures were finally diluted to an OD_600 nm_ of 0.05 in CDM adjusted to the pH and polyamine concentration of interest. These last cultures were incubated at 37 °C for 16 h in microtitre plates (Greiner Bio-One; Cellstar) (300 µl culture volume per well) in an Eon thermoregulated spectrophotometer plate reader (BioTek Instruments). The OD_600 nm_ was measured every hour after double orbital shaking of the plate for 5 s. The reported OD_600 nm_ is the average OD of three wells inoculated with the same culture. Three independent experiments were realized for all strains and for all tested conditions.

### Bioinformatics analysis

To identify *S. agalactiae* homologues of enzymes involved in the known putrescine, spermidine and spermine biosynthesis pathways, the non-redundant protein sequences of *S. agalactiae *, available at the NCBI database on the 5 July 2019, were blasted with the amino acid sequence of the constitutive (SpeC) and inducible (SpeF) ornithine decarboxylase, the inducible arginine decarboxylase (AdiA) and the l-methionine adenosyltransferase (MetK) of *E. coli* (accession nos. NP_417440, NP_415220, NP_418541.2 and NP_417417), with the arginine decarboxylase (accession no. ABJ55263.1), the agmatine deiminase (accession no. ABJ54341.1), the N-carbamoyl putrescine amidohydrolase (accession no. ABJ55190), the carboxyspermidine dehydrogenase (accession no. ABJ55062.1), the carboxyspermidine decarboxylase (accession no. ABJ53691) and the spermidine synthase (accession no. ABJ54021.1) of *S. pneumoniae *, with the *S*-adenosyl-methionine decarboxylase (SpeD), the aspartate kinase III, the aspartate-β-semialdehyde dehydrogenease, the agmatinase (SpeB) and the spermine synthase of *Bacillus subtilis* (accession nos. NC_00964.3, NP_388261.1, NP_389557.1, NP_391629 and QBJ65844, respectively), as well as with the agmatine aminopropyltransferase and the aminopropylagmatine ureohydrolase of *Thermus thermophilus* (accession nos. WP_011172918 and WP_011228458.1. The sequences of *S. agalactiae* genomes available as whole genome contigs or as complete genome sequences at the NCBI database were also blasted with the nucleotide coding sequences of the *S. pneumoniae* enzymes mentioned above.

To identify σ^70^ transcriptional promoters, the sequence of the intergenic regions between *folK* and *murB*, and *potD* and *clc*, of strain A909 were analysed with the BProm software from the SoftBerry suite (http:// www.softberry.com/berry.phtml?topic=bprom&group=programs& subgroup=gﬁndb).

Rho-independent transcriptional terminators were searched with the Arnold program (http://rssf.i2bc.paris-saclay.fr/toolbox/arnold/) with the sequences of all intergenic regions and with the entire coding sequence of *murB*, *potA*, *potB*, *potC*, *potD*, *clc* and *araC*.

Prediction of transmembrane helices in PotB and PotC was performed by the TMHMM Server of the Center for Biological Sequence Analysis at the Technical University of Denmark (http://www.cbs.dtu.dk/services/TMHMM/).

The presence of a signal peptide and the location of its cleavage sites in PotD was predicted with the SignalP 5.0 server (http://www.cbs.dtu.dk/services/SignalP/).

The synteny of the *folK-murB-potABCD-clc* region in streptococcal species was compared via the SyntTax web server (https://archaea.i2bc.paris-saclay.fr/synttax/).

### Survival of *S. agalactiae* to peroxidase-induced oxidative and acidic stress

To measure the ability of *S. agalactiae* strains to survive in CDM at pH 4.0, the bacteria were cultured at 37 °C (without agitation) in non-buffered TH broth at pH 7.4 to the beginning of the stationary phase of growth. This culture was then centrifuged, washed with non-buffered CDM at pH 7.4, and suspended to an OD_600nm_ of 0.005 in 40 ml of the same medium. After an overnight incubation at 37 °C without agitation, 10 ml aliquots of this culture were transferred to Falcon tubes, which were centrifuged for 5 min at 5000 ***g***. Bacterial pellets were suspended in either 1 ml CDM buffered at pH 4.0 with a 100 mM mix of Na citrate and citric acid without the presence of polyamines or in 1 ml of the same medium containing 1 mM spermidine, spermine or putrescine. These suspensions were then incubated at 37 °C (without agitation) for 6 h. Viable cell counts of the bacteria were performed immediately after suspension of the pellets (t_0_) and at suitable time intervals thereafter. To this end, serial dilutions were performed in TH broth at pH 7.4. Then 100 μl of each of these dilutions was immediately spread three times onto TH agar plates, which were incubated at 37 °C for 24 h. All survival experiments were performed at least three times. Results are expressed as the percentage of survivors [(number of viable bacteria at the tested condition divided by the number of viable bacteria at t_0_)×100].

To compare the sensitivity of the *potABCD* mutant and the wild-type strains to peroxidase-induced oxidative stress, *S. agalactiae* strains were grown to an OD_600 nm_ of 0.6 in TH broth and then exposed to different concentrations of H_2_O_2_ (1, 5 or 20 mM).

To test if polyamines are involved in the survival of *S. agalactiae* strains submitted to peroxidase-induced oxidative stress, *S. agalactiae* cultures were grown in TH broth to the beginning of the stationary phase. Each culture was then centrifuged, washed with non-buffered CDM at pH 7.4, and suspended to an OD_600 nm_ of 0.005 in 10 ml of the same medium and grown overnight at 37 °C without agitation. These last cultures were finally diluted to an OD_600 nm_ of 0.05 in CDM at pH 7.4, and grown until an OD_600 nm_ of 0.6 (exponential phase). Bacterial cultures (10 ml samples) were then harvested and exposed to peroxidase-induced oxidative stress after suspension in the same volume of CDM containing either 5 mM H_2_O_2_, 5 mM H_2_O_2_ and 1 mM spermidine, 5 mM H_2_O_2_ and 1 mM spermine, or 5 mM H_2_O_2_ and 1 mM putrescine. Viable cell counts of the bacteria were performed immediately after the suspension of the pellets (t_0_) and at suitable time intervals thereafter. To this end, serial dilutions were performed in TH broth at pH 7.4. Then 100 μl of each of these dilutions was immediately spread onto TH agar plates, which were incubated at 37 °C for 24 h. All survival experiments were performed at least three times. The results are expressed as the percentage of survivors [(number of viable bacteria at the tested condition divided by the number of viable bacteria at t_0_)×100].

### Expression of *potABCD* during acidic and peroxidase-induced oxidative stress

To quantify the expression of the *potABCD* operon during acidic stress, strain A909 was grown in Falcon tubes containing 10 ml TH broth to the beginning of the stationary phase of growth. Each culture was then centrifuged, washed with non-buffered CDM at pH 7.4, suspended to an OD_600 nm_ of 0.005 in 10 ml of the same medium, and grown overnight at 37 °C without agitation. These last cultures were finally diluted to an OD_600 nm_ of 0.05 in CDM at pH 7.4, and grown to an OD_600 nm_ of 0.6 (exponential phase). Bacterial cells (10 ml samples) were then harvested, centrifuged, and suspended either in 10 ml CDM buffered at pH 5.5 with 100 mM MES or in CDM buffered at pH 4.0 with a mix of Na citrate and citric acid. These media were either supplemented or not with 1 mM spermidine, spermine or putrescine. The bacteria were exposed to the acidic stress for 30 min. A control culture grown at pH 7.4 in CDM without acidic stress was treated similarly. All cultures were then centrifuged at 5000 ***g***. Bacterial pellets were collected and then stored at −80 °C until RNA extraction. This experiment was repeated three times from three independent cultures.

To determine the expression of *potA* during peroxidase-induced oxidative stress by quantitative reverse transcriptase (qRT-PCR), the same treatment was performed as described for the peroxidase-induced oxidative stress survival assay. Thus, the wild-type cultures were collected after either 20 or 60 min of exposure to 5 mM H_2_O_2_, in the absence and in the presence of each type of polyamine. A control culture grown at pH 7.4 in CDM without any stress was always collected at the same time. Cultures were then centrifuged at 5000 ***g***. Bacterial pellets were collected and then stored at −80 °C until RNA extraction. This experiment was repeated three times from three independent cultures.

### Nucleic acid manipulations

Standard nucleic acid manipulation techniques were carried out as described previously [45]. *S. agalactiae* genomic DNA and RNA purifications were performed as previously described [40]. Plasmids were purified from *E. coli* with a NucleoSpin Plasmid kit (Macherey‐Nagel), according to the manufacturer’s instructions. Nucleic acid concentrations were measured with a NanoDrop Lite Spectrophotomer (Thermo Scientific). The ratio of absorbance at 260 and 280 nm was used to check the purity of nucleic acids. Bacteria were transformed by electroporation with the Micropulser apparatus (Bio-Rad) and the Ec2 conditions (2.5 kV), as described by Dower for *E. coli* and by Ricci for *S. agalactiae* [46, 47].

### Amplification of nucleic sequences by PCR, by RT-PCR and by qRT-PCR

PCR was carried out with the Applied Biosystem 2720 Thermal cycler using Q5 High-Fidelity DNA polymerase (New England Biolab) for cloning or sequencing or OneTaq polymerase (New England Biolab) for analytical PCR. For cloning or sequencing, the resulting PCR fragments were further purified with a NucleoSpin Gel and PCR clean-up kit (Macherey-Nagel) or with a NucleoSEQ kit (Macherey-Nagel), according to the manufacturer’s instructions. The oligonucleotides (Sigma-Aldrich) used in this study are listed in Table 2.

**Table 2.** Primers used in this study

Name*	Sequence†	Location‡
**Primers used for RT-PCR**		
SK6_fw_	ACCCTTCTTGTGGTTCTGTGTT	nt 647 to 668 of *murB*
SK7_rv_	TCTGAGTTAGGTGTTGAACTACTCTTCTT	nt 107 to 79 of *potD*
SK8_fw_	TGGTTATGCGACCCCTAATCT	nt 894 to 914 of *potD*
SK9_rv_	CCTATTATCACTGCAGCGATAATACTAGT	nt 227 to 199 of *clc*
SK10_rv_	CAAAAACCGTCATATGTGGAAAC	nt 292 to 270 of *potA*
SK16_fw_	GCGTGAGACATGAGCACTG	nt 248 to 266 of *folK*
SK17_rv_	GCGATAGTTCAAGGCGATTC	nt 136 to 117 of *murB*
SK29_fw_	ATTAAGCAGGGTGGGACG	nt 232 to 249 of *potD*
SK30_rv_	AAATGCTTATTCCCATCAAGC	nt 749 to 729 of *potD*
SK32_fw_	AAAGTCGCGCAAATTTCC	nt 97 to 114 of *folk*
SK33_rv_	CTTCGTCATATACGGATGGG	nt 360 to 341 of *folk*
**Primers used for qRT-PCR**		
SK35_fw_	GGCGAGTACAGGCGATATTT	nt 174 to 193 of *potA*
SK36_rv_	AGCTCGCTTTTCAAACCCTT	nt 402 to 383 of *potA*
OLM321_fw_	CTGGTGGTCGTGCTTTGAAA	nt 551 to 570 of *recA*
OLM322_rv_	TATGCTCACCAGTCCCCTTG	nt 634 to 615 of *recA*
**Primers used for the deletion of** ***potABCD***		
OAH295_fw_	GTTTTA**GGATCC** GCGAGTAATATTATCGTTAGAGATGG	nt 190 to 215 of *murB*
OAH305_rv_	T**GGTCTC**GATTACTTTATAATAGCGACTCACCGATA	nt – 45 upstream of *potA* to nt 882 of *murB*
OAH306_fw_	T**GGTCTC**GTAATTGGGATGCTAT TGG	nt 339 to 356 of *potD*
OAH307_rv_	TCATTG**GGTACC** CCAAGCCATTTTTGACCC AAG	nt 1028 to 1008 of *potD*
**Primers used specifically for sequencing**		
SK3_fw_	ATGTATCGTCAACAACCTATATTTTTGG	nt 532 to 559 of *folK*
SK5_rv_	TTGAGTTCACCCTGTGGTGT	nt 476 to 457 of *murB*
SK6_fw_	ACCCTTCTTGTGGTTCTGTGTT	nt 647 to 668 of *murB*
SK8_fw_	TGGTTATGCGACCCCTAATCT	nt 894 to 914 of *potD*

*fw, forward primer; rv, reverse primer.

†Tails containing a restriction site (in bold) are underlined.

‡Nucleotide (nt) position with respect to the first coding nt of the gene of interest or to a polylinker restriction site.

**Fig. 2.**
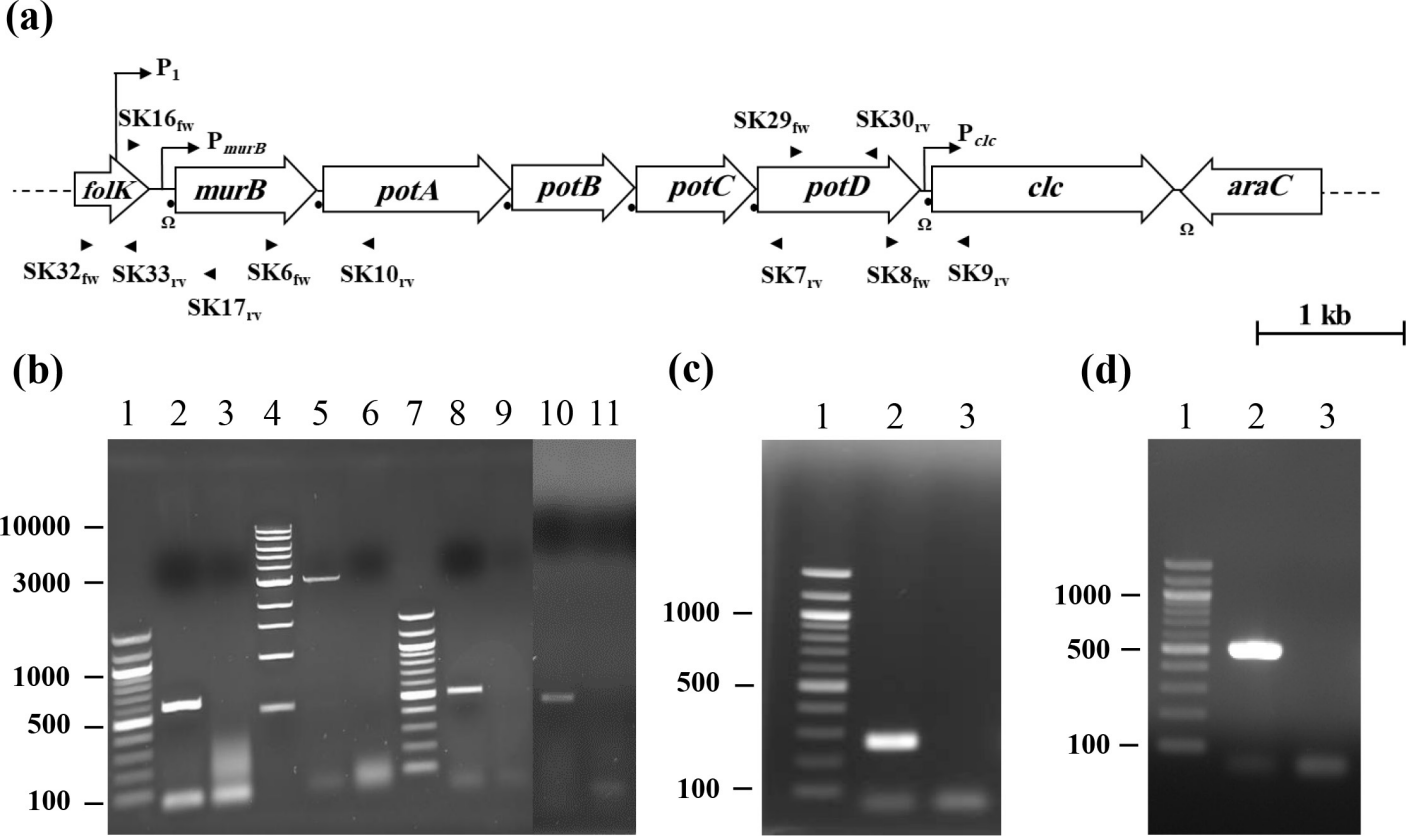
Transcriptional analysis of the *potABCD* region of *Streptococcus agalactiae* A909. Putative transcriptional promoters (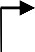) and rho-independent terminators (Ω) were identified *in silico* by the BPROM and the Arnold software, respectively. ORFs (open arrows), ribosome binding sites (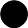) and primers (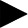) used in RT-PCR experiments are indicated in the schematic representation of the *folK-araC* region of *S. agalactiae* (**a**). Co-transcripts between *murB* and *potA* (lane 2b), between *murB* and *potD* (lane 5b), between *potD* and *clc* (lane 8b), and between *folK* and *murB* (lane 10b) were revealed by RT-PCR performed with primers annealing in *murB* and *potA* (SK6_fw_/SK10_rv_), in *murB* and *potD* (SK6_fw_/SK7_rv_), in *potD* and *clc* (SK8_fw_/SK9_rv_), and in *folK* and *murB* (SK16_fw_/SK17_rv_). The *folK* (lane 2c) or *potD* (lane 2d) transcripts were revealed by RT-PCR performed with primers annealing in *folK* (SK32f_w_/SK33_rv_) or in *potD* (SK29_fw_/SK30_rv_), respectively. RT-PCRs were performed in the absence of reverse transcriptase to check for DNA contamination (lanes 3b, 6b, 9b, 11b, 3c and 3d). Amplification products were electrophoresed in a 1 % agarose gel containing ethidium bromide and visualized under UV light (260 nm). Molecular weight markers (Quick-Load Purple, 100 bp and 1 kb DNA Ladder; New England Biolabs) of the indicated sizes are in lanes 1b, 7b, 1c or 1d and 4b, respectively.

For RT-PCR and qRT-PCR, the RNAs were reverse transcribed as previously described [40]. For RT-PCR, cDNAs were amplified by PCR with appropriate primers (Fig. 2a, Table 2), as described above for PCR amplification of DNA. Control RT-PCRs, omitting reverse transcriptase, were performed to check for DNA contamination of the RNA preparation.

For qRT-PCR, primers were selected with Primer3web software (https://bioinfo.ut.ee/primer3/) in order to generate 100 to 300 bp amplicons (Table 2). qRT-PCRs were performed in a 20 µl reaction volume containing 40 ng of cDNA, 0.5 µl of gene-speciﬁc primers (10 µM) and 7.5 µl LightCycler 480 SYBR Green I Master 2× (Roche, code no. 04707516001). PCR ampliﬁcation, detection and analysis were realized with the Bio-Rad CFX Connect Real-Time PCR detection system and Bio-Rad CFX Maestro software. PCR conditions included an initial denaturation step at 95 °C for 5 min, followed by a 40-cycle ampliﬁcation (95 °C for 10 s, 60 °C for 20 s and 72 °C for 20 s). The speciﬁcity of the ampliﬁed product and the absence of primer dimer formation were veriﬁed by generating a melting curve (65–98 °C, continuous increase). The cycle threshold (Ct) was deﬁned for each sample. Expression levels of the tested genes were normalized using the *recA* gene (primers OLM321 and OLM322) of *S. agalactiae *. Transcript levels of *recA* did not vary under our experimental conditions (Table 2). The fold change in the transcript level was calculated using the following equations: ΔCt=Ct (target gene) – Ct (*recA* gene); ΔΔCt=ΔCt (reference condition) – ΔCt (test condition); relative quantification (RQ)=2^−ΔΔCt^. Each assay was performed in triplicate and repeated with at least three independent RNA samples.

### DNA sequencing

PCR products were sequenced on both strands using the Big Dye Terminator v3.1 cycle sequencing kit from Applied Biosystems and the ABI Prism 310 Genetic Analyzer.

### Construction of *potABCD* deletion mutant

*S. agalactiae* A909Δ*potABCD* is a non-polar mutant of strain A909 deleted by allelic exchange of a DNA region beginning five nucleotides after the stop of *murB* and ending 338 nucleotides after the start of *potD*. Upstream and downstream regions of the deleted region were amplified by PCR with primers OAH295_fw_/OAH305_rv_ and OAH306_fw_/OAH307_rv_, respectively. These amplified fragments were cut by *Bsa*I, and a recombination cassette, consisting of a fusion between these two regions, was obtained by splicing‐by‐overlap‐extension PCR with primers OAH295_fw_ and OAH307_rv_ (Table 2). To carry out chromosomal gene inactivation, the overlap‐extension fragment was hydrolysed by *Bam*HI and *Kpn*I, and cloned into the *Bam*HI/*Kpn*I sites of the thermosensitive shuttle plasmid pG +host1 [48]. The recombinant plasmid was electroporated in *E. coli* for amplification, purified and finally electroporated in strain A909. Allelic exchange was performed as described by Biswas [48]. Deletion of the *potABCD* region of *S. agalactiae* A909 was confirmed by sequencing with primers SK3_fw_, SK5_rv_, SK6_fw_ and SK8_fw_ (Table 2).

### Determination of the intracellular polyamine content

Intracellular polyamine content was determined as described in the literature [49, 50]. In brief, aliquots from bacterial cultures were pelleted. Then, 200 mg (wet weight) of bacteria was washed four times with PBS and suspended in 1 ml of 0.2 M perchloric acid. They were subsequently disrupted by sonication and centrifuged for 10 min at 12 000 ***g*** (4 °C). The supernatant was collected and 200 μl of the extract was dansylated by the addition of 0.4 ml of a solution of dansylchloride (30 mg dansylchloride ml^-1^ acetone) and 50 mg Na_2_CO_3_.10H_2_0. After incubation for 16 h in the dark (ambient temperature), 0.1 ml of a proline solution was added (100 mg ml^−1^ H_2_O Milli-Q^R^) and the extract was incubated again for 30 min. Dansylated polyamines were extracted with 700 µl of toluene. Dansylated toluene extracts (40 µl) were then determinated by TLC on silica gel 60G plates (Merck). The dansylated polyamines were separated by development in ethylacetate/cyclohexane (2 : 3, v/v) followed immediately by spraying the TLC plate with triethanolamine/cyclohexane (1 : 4, v/v) to enhance and stabilize fluorescence. Similarly, 200 µl of a 1 mM solution of spermine, spermidine or putrescine in Milli-Q^R^ water was dansylated, extracted with toluene and 40 µl of each extract was assessed by TLC. After drying, spots were visualized under Wood light and photographed.

### Statistical analyses

Data are presented as the mean±sd for three independent experiments. An unpaired Student’s *t*-test was used to determine the significance of the differences between means [51].

## Results and discussion

### The putrescine, spermidine and spermine biosynthesis pathways are not present in *S. agalactiae*

We searched, by blastP analysis, if *S. agalactiae* possesses homologues of enzymes involved in the known putrescine, spermidine and spermine biosynthesis pathways (Fig. 1). Only three enzymes (aspartate kinase, aspartate β semialdehyde dehydrogenase and l-methionine adenosyl transferase) involved in one or the other first steps of spermidine or spermine biosynthesis were encoded by all strains of *S. agalactiae *. These three enzymes are also involved in the biosynthesis of several amino acids and are essential for *S. agalactiae* (e.g. SAK_0414, SAK_0954 and SAK_1141 in https://www.genome.jp/kegg-bin/show_organism?org=sak). One (DK-PW-092) of the 1018 analysed strains also encodes an agmatine deiminase (gene WA34_16675) and an *N*-carbamoylputrescine amidohydrolase (gene WA34_ 16685). In the genus *Streptococcus *, the presence of polyamine biosynthesis pathways was searched by others in three other species. All the genomes of *S. pneumoniae* and the two completely sequenced genomes of *Streptococcus oralis* encode the enzymes necessary for the polyamine biosynthesis pathways IIp, Is and IIs (Fig. 1). However, these three pathways were found in only two of all the 30 completely sequenced genomes of *S. suis* and in two of the three completely sequenced genomes of *S. mitis* [11, 19]. It is not known if the need of certain streptococcal species for a polyamine biosynthesis pathway reflects their lifestyle and their ability to survive at certain stages in polyamine-free environments.

**Fig. 3.**
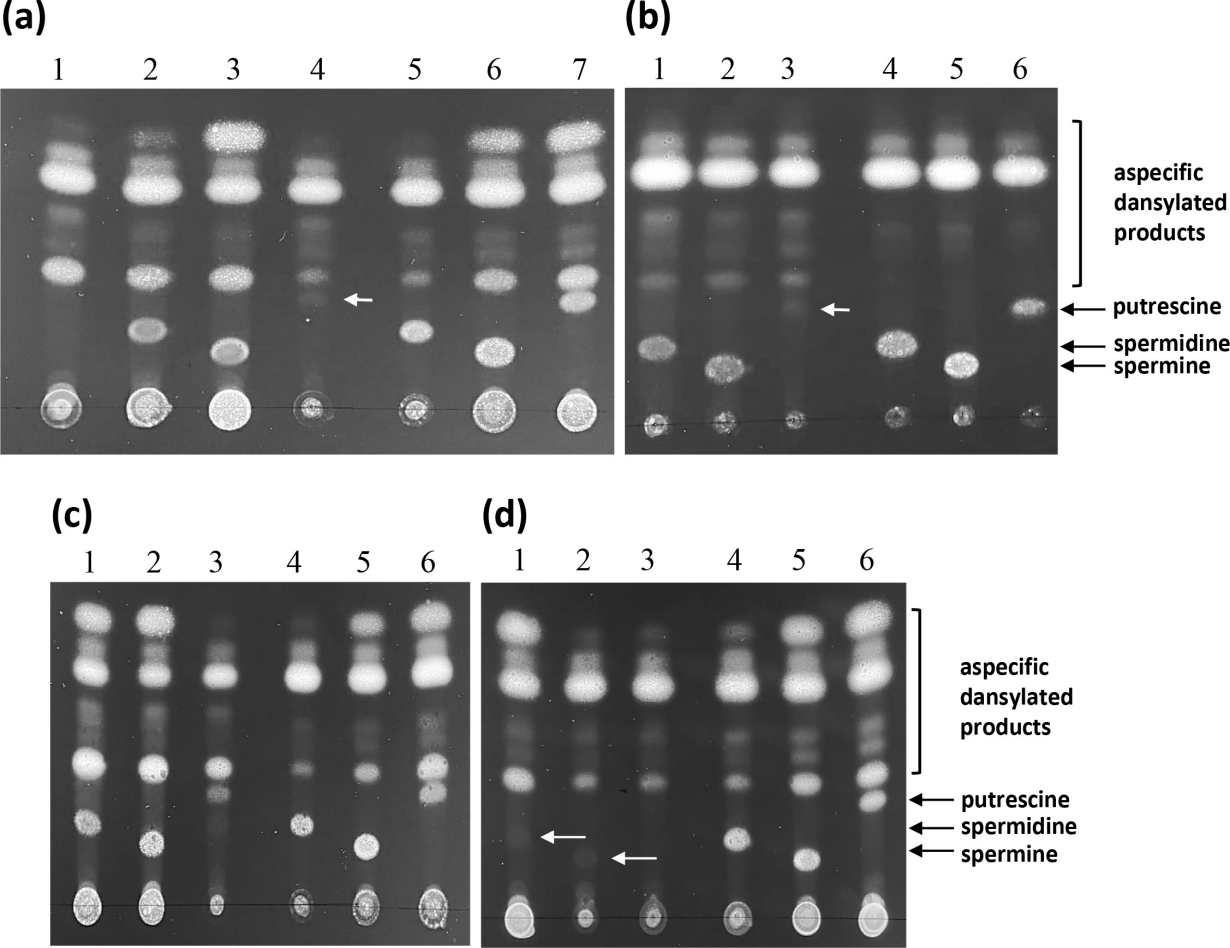
Intracellular polyamine content of *S. agalactiae *. *S. agalactiae* wild-type strain (a and c) or Δ*potABCD* mutant (b and d) were grown either to the beginning of the stationary phase (a and b) or to an OD_600 nm_ of 0.6 (exponential growth phase, c and d) in a chemically defined medium at pH 7.4 in the absence (lane 1a) and in the presence of 1 mM spermidine (lanes 2a, 1b, 1c and 1d), 1 mM spermine (lanes 3a, 2b, 2c and 2D) or 1 mM putrescine (lanes 4a, 3b, 3c and 3d). In (c) and (d), bacteria were then exposed to 5 mM H_2_O_2_ for 60 min Bacterial extracts were dansylated, separated by TLC and photographed under Wood light. Dansylated standards of spermidine (0.2 µg; lanes 5a, 4b, 4c and 4d), spermine (0.2 µg; lanes 6a, 5b, 5c and 5d) and putrescine (0.1 µg; lanes 7a, 6b, 6c and 6d) were deposited in each TLC plate. The white arrows indicate the intracellular presence of a small quantity of putrescine, spermine or spermidine.

As homologues of most of the enzymes involved in the known biosynthesis pathways of putrescine, spermidine and spermine are absent in *S. agalactiae *, this bacterium should not be able to synthesize these polyamines, and presumably acquired them from the environment. We verified this hypothesis by TLC analyses performed on the intracellular polyamine content of bacteria grown in the absence or in the presence of polyamines. Fig. 3(a) shows that *S. agalactiae* is unable to biosynthesize spermine, spermidine or putrescine (lane 1a), whereas it is able to strongly transport spermidine and spermine (lanes 2a and 3a) and slightly transport putrescine (lane 4a, white arrow) at pH 7.4.

### Prevalence of the *potABCD* genes of *S. agalactiae* and characteristics of the transporter

As almost all bacteria can import extracellular polyamines by the ABC transporter *potABCD*, we searched the prevalence of the *potABCD* genes in *S. agalactiae* strains. To that end, blastN analyses with the nucleotide sequence of *potABCD* of strain A909 were performed in the non-redundant nucleotide collection of *S. agalactiae* sequences, available at the NCBI database. This operon is conserved in all the completely sequenced genomes of *S. agalactiae* (130 strains at the time of our analysis). We also screened this databank by tblastN with the PotA, PotB, PotC and PotD protein sequences of strain A909. In 90.8, 93.1, 97.0 and 99.2% of these strains, the PotA, PotB, PotC or PotD sequences are identical to those of strain A909, respectively. The few non-identical PotA, PotB or PotC proteins were translated from nucleotide sequences of pseudogenes. Non-identical PotA proteins contain only one mismatch with respect to the sequence of strain A909. Seventeen per cent of these mismatches concern similar amino acids.

The amino acid sequence of PotA has the characteristic motifs of the nucleotide binding proteins [52]. It contains the two ATP/GTP binding site motifs Walker A and Walker B at positions 40–47 and 162–169, respectively. The characteristic ABC transporter family signature, with one mismatch, was also identified as correctly located (position 162–169) between the two Walker motifs. PotA also has at a correct location (position 192–197), the Linton and Higgins motif, containing at the fifth position the highly conserved histidine implicated in the function of the ABC transporters. PotB and PotC also have characteristic motifs of membrane spanning proteins of ABC transporters. PotB and PotC have 24 % identical and 16 % similar amino acids in common. Both of them are integral transmembrane proteins predicted to be composed of six transmembrane domains by the THMM server. PotB and PotC contain also the ABC transporter integral membrane type-1 domain (PS50928) at positions 57–259 and 58–246, respectively. The signalP server predicts the existence of a signal peptide (sec/SPI) in the substrate binding protein PotD, with a cleavage site between position 31 and 32. Substrate binding proteins of Gram-positive ABC transporters are generally lipoprotein [52]. However, no lipid attachment site could be identified in PotD. We also searched for the presence of other domains involved in the anchoring of proteins to the surface of Gram-positive bacteria: LPXTG sequences for binding to the peptidoglycan by a sortase or choline-binding domains for the attachment to choline residues of lipoteichoic acids. None of them could be identified in PotD. This protein may thus be anchored to the cytoplasmic membrane by an as yet unidentified mechanism. A similar situation was described for PotD of *S. pneumoniae* [53]. PotD of *S. agalactiae* possesses the bacterial spermidine/putrescine binding motif described by Shah and collaborators [54]. This binding fold is composed of two globular subdomains connected by a flexible hinge and bind their ligand in the cleft between these domains in a manner resembling a Venus flytrap [55].

### Transcriptional analysis of the *potABCD* region of *S. agalactiae* A909

The *potABCD* genes of *S. agalactiae* are transcribed in the same direction as the *fol* genes, *murB* and *clc*, suggesting that they are co-transcribed (Fig. 2a). Most of these genes are separated by short intergenic sequences (48, 11 and 2 nt between *murB* and *potA*, *potA* and *potB*, and *potC* and *potD*, respectively) or possessed overlapping stop and start codons (*potB* and *potC*). The *folK–murB* and *potD–clc* intergenic regions are 143 and 108 nt long, respectively. Correctly conserved and positioned ribosome binding sites were identified upstream of all ORFs of the *murB–clc* region. We previously identified σ^70^ transcriptional promoters inside the *folK* gene (P_1_: −10 box, TTGAATTAT; −35 box, TAGAGA) and in the *folK–murB* intergenic region (P*_murB_*: −10 box, TGGTATAAT; −35 box, TCGTCA). Both of them were shown to be involved in the transcription of *murB* [40]. We here also identified putative σ^70^ transcriptional promoters in the *potD–clc* intergenic region (P*_clc_*; −10 box: TGTTAAAAT, −35 box: TTCCTA). Potential Rho-independent transcriptional terminators were also identified in the *folK–murB* intergenic region (position −73 to −36 upstream of *murB*; Δ*G*=−5.3 kcal), in the *potD-clc* intergenic region (position −104 to −62 upstream of *clc*; Δ*G*=−9.7 kcal), and downstream of *clc* (position +22 downstream of *clc* to nt 920 of *araC*; ∆*G*=−10.4 kcal). By RT-PCRs performed with reverse primers annealing either in *potA* or in *potD* (SK10_rv_ or SK7_rv_, Table 2, Fig. 2a), and a forward primer annealing in *murB* (SK6_fw_, Table 2, Fig. 2a), we here showed that *murB* is also co-transcribed with *potABCD* (Fig. 2, lanes 2b and 5b, respectively). Similarly, by using the SK9 reverse primer annealing in *clc* and the SK8 forward primer annealing in *potD* (Table 2, Fig. 2a), we showed that *potD* is co-transcribed with *clc* (Fig. 2, lane 8b). We previously showed that *folK* is co-transcribed with *murB* in strain SA87 [40]. By using the SK17 reverse primer annealing in *murB* and the SK16 forward primer annealing in *folK* (Table 2, Fig. 2a), we confirm this fact in strain A909 (Fig. 2, lane 10b). As co-transcription of *folK* and *murB* and of *potD* and *clc* were demonstrated, the role of the two hairpin structures as transcriptional terminators is questionable; these structures could perhaps be attenuators or binding sites for a regulator protein. It is of note that an attenuator structure was identified in the leader sequence of the *potABCD* operon of *Haemophilus somnus* and of *Pasteurella multocida* [56]. We have thus compared the level of *folK* and of *potD* transcripts with those of *folK–murB* and *potD–clc* co-transcripts. To that end, RT-PCR was performed with reverse and forward primers annealing in *folK* (SK33_rv_ and SK32_fw_) and in *potD* (SK30_rv_ and SK29_fw_) (Table 2, Fig. 2a). Although this experiment is rather qualitative, amplification of the *folK–murB* (Fig. 2, lane 10b) and *potD–clc* (Fig. 2, lane 8b) co-transcripts is lower than that of *folK* (Fig. 2, lane 2c) or *potD* (Fig. 2, lane 2d), suggesting that the two hairpin structures arrest some of the upstream transcripts.

In conclusion, although the *murB–potABCD* and the *clc* genes are also likely transcribed from their own P*_murB_* and P*_clc_* promoters, the above results suggest that strain A909 synchronizes the biosynthesis of folate and peptidoglycan with the transport of a polyamine by PotABCD and with the resistance to acidic stress. An RNA-sequencing experiment conducted on strain NEM316 of *S. agalactiae* indicated that the P*_murB_* and the P*_clc_* promoters are functional and that the *murB–potABCD* and *clc* transcripts are the major transcripts of the *folK–clc* region [57]. As the *murB* and *potABCD* genes are also co-transcribed in *S. pneumoniae* and *S. suis,* it appears that it is particularly important for streptococcal species to coordinate these two processes involved in cell wall synthesis [19, 41]. It is nevertheless of note that although the synteny of the *murB* and *potABCD* genes is conserved in the entire genus *Streptococcu* s, this is not the case for the complete *folK–murB–potABCD–clc* region. We compared the synteny of this region in the genomes of 676 streptococal strains representative of the genus *Streptococcus* and found that the entire *folK–clc* region is only conserved in species belonging to the pyogenic group (* S. agalactiae *, *S. dysgalactiae* subsp. *equisimilis *, *S. equi* subsp. *equi *, *S. equi* subsp. *zooepidemicus *, *S. canis *) and in non-pyogenic species of the Bovis group (* S. equinus *, *S. gallolyticus *, *S. infantarius *, *S. lutetiensis *, *S. macedonicus *, *S. pasteurianus *). In the non-pyogenic species *S. suis *, the synteny is only conserved from *potA* to *clc*. However, Liu and collaborators were not able to show a co-transcription between *potABCD* and *clc* in this species [19] .

### No requirement of polyamines and of the PotABCD transporter for the growth of *S. agalactiae* at pH 7.4

**Fig. 4.**
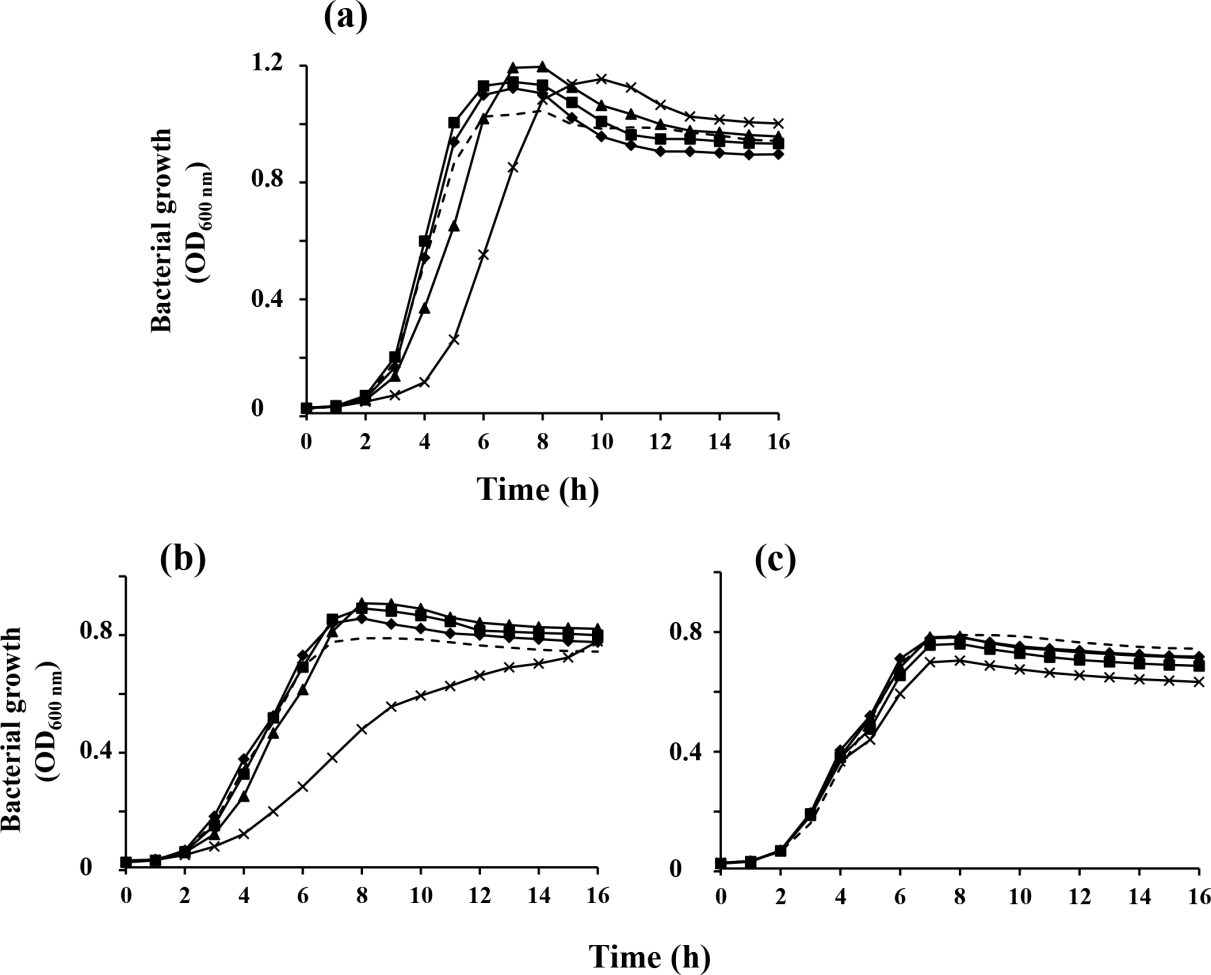
Growth of *S. agalactiae* strain A909 in a chemically defined medium containing different concentrations of polyamines. *S. agalactiae* A909 was grown in a chemically defined medium buffered at pH 7.4 with 100 mM HEPES in the absence of polyamines (---) or in the presence of various amounts (♦, 0.5 mM; ■, 1 mM; ▲, 2 mM and ×, 5 mM) of spermidine (**a**), spermine (**b**) or putrescine (**c**). These cultures were incubated for 16 h at 37 °C without agitation in microtitre plates (300 μl culture volume per well) in an Eon thermoregulated spectrophotometer plate reader. The OD_600 nm_ was measured every hour after double orbital shaking of the plate for 5 s. The reported OD_600 nm_ is the average OD of three wells inoculated with the same culture. Three independent experiments were realized for all tested conditions. Standard deviations were always less than 10 %.

Since *S. agalactiae* seems to be obligated to acquire polyamines from the environment, we tested if the PotABCD transporter and exogenous polyamines are required to sustain growth of this bacterium. We first grew *S. agalactiae* strain A909 in a chemically defined medium containing various concentrations of spermine, spermidine or putrescine. As polyamines are very basic components, this medium was buffered at pH 7.4 with 100 mM HEPES. Concentrations of spermidine or spermine of 1 mM or below have only a very slight positive impact on the growth of *S. agalactiae *, but higher concentrations have an inhibitory impact (Fig. 4a, b). Putrescine has no effect on the growth of *S. agalactiae* until a concentration of 5 mM, when it becomes slightly inhibitory (Fig. 4c). We then constructed a deletion mutant of *potABCD* to ensure that traces of polyamines eventually contaminating our minimal medium are not transported by the PotABCD transporter, thus allowing an identical growth of the wild-type strain in the presence or not of each of the polyamines tested. This assumption is, however, invalid as strains A909 and A909∆*potABCD* grow identically either in the rich TH medium or in the chemically defined medium in the absence of added polyamines (Fig. S1a, b, available in the online version of this article). No difference between the growth of the wild-type strain and of the *potABCD* deletion mutant in a chemically defined medium containing 1 mM spermidine, 1 mM spermine or 1 mM putrescine were also noted (Fig. S1c–e, respectively). Furthermore, TLC analyses indicate that both the wild-type strain and the Δ*potABCD* mutant efficiently import spermidine and spermine and putrescine faintly (Fig. 3a, b, respectively). Therefore, the *potABCD* transporter is not the main and only importer of polyamine in *S. agalactiae *.

In conclusion*, S. agalactiae* does not have an absolute need of exogenous polyamines in its environment, does not encode any specific enzymes involved in the synthesis of polyamines, has an undetectable content of intracellular polyamines when grown in the absence of polyamines, and does not need the PotABCD transporter for efficient *in vitro* growth in physiological conditions at pH 7.4. Bacterial species thus have different comportments with respect to polyamine requirement because, for optimal growth, *S. pneumoniae,* in which the polyamine biosynthesis pathways were inhibited, or strains of *S. suis* devoid of the polyamine biosynthesis pathways IIp, Is and IIs, show delayed growth after the deletion of genes encoding the PotABCD transporter [19, 58]. Supplementation of growth media with polyamines was also shown to enhance the growth of certain bacteria such as *E. coli* mutants, which cannot synthesize polyamines, or *Legionella pneumophila,* which does not possess the enzymes required for polyamine biosynthesis [59, 60].

Exogenous polyamine supplementation greater than 1 mM inhibited *S. agalactiae* in a dose-dependent manner (Fig. 4 and data not shown). The bactericidal effect of certain concentrations of polyamines was reported for several bacterial species, and particularly in *Staphylococcus aureus *, which is hypersensitive to these compounds, even at a physiological concentration [19, 61]. The reason for this inhibitory effect is not completely understood. Several reports have described a relationship between the toxicity of polyamines towards *Staphylococcus aureus* and an increase of pH of the medium. It was suggested that polyamine toxicity is inversely proportional to the net cationic charge of polyamines since they become sequentially deprotonated at elevated pH [61, 62]. It was also found that *Staphylococcus aureus* polyamine sensitivity is mediated by menaquinone but is independent of respiration [61]. In *E. coli *, to avoid spermidine toxicity, high concentration of spermidine inhibit the polyamine ABC transporter, through the interaction of spermidine with PotA [28]. In addition, PotD is a retroactive regulator of the transcription of *potABCD* [63]. It is not known if these mechanisms exist and are efficient in *S. agalactiae *.

### Spermine and spermidine induce the transcription of *potABCD*

**Fig. 5.**
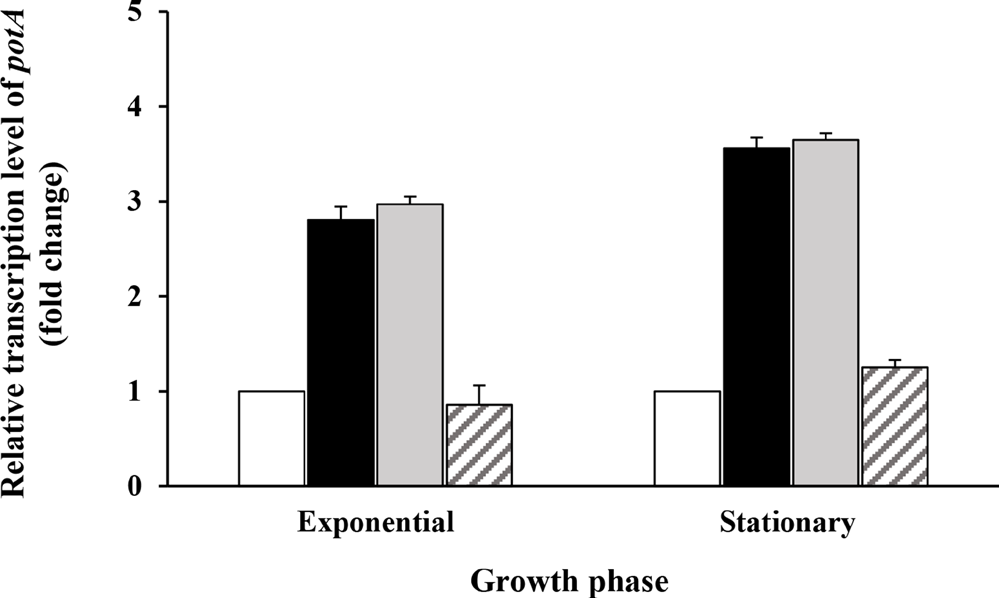
Induction of *potABCD* by polyamines at pH 7.4. qRT-PCR of *potA* transcripts was performed on RNA extracts of *S. agalactiae* A909 grown to an OD_600 nm_ of 0.6 (exponential phase) or of 1.2 (stationary phase) at 37 °C in a chemically defined medium buffered with 100 mM HEPES at pH 7.4, in the absence of polyamines (white bars) or in the presence of 1 mM spermidine (black bars), 1 mM spermine (grey bars) or 1 mM putrescine (striped bars). Transcript levels of *potA* were normalized against *recA* transcript levels. Gene expressions are presented as fold change with regard to the level of *potA* transcripts during growth of *S. agalactiae* in the absence of polyamines. Results are presented as means±sd of three independent experiments.

We tried to correlate the transport of polyamines with the level of transcription of the *potABCD* operon. To that end, we quantified *potA* mRNA by qRT-PCR during growth of the bacteria in a chemically defined medium containing or lacking 1 mM spermine, spermidine or putrescine. As shown in Fig. 5, spermidine and spermine induce the expression of *potABCD*, during both the exponential (induction factor of 2.80 and 2.96, respectively) and the stationary phase (induction factor of 3.55 and 3.64, respectively) of growth. In contrast, putrescine does not have this effect. These data are in agreement with the intracellular polyamine content of cells grown in media with spermine, spermidine or putrescine (Fig. 3a).

Similarly, in *S. suis *, spermine and spermidine were found to induce the expression of the *potABCD* operon, but related experiments in *S. pneumoniae* gave different results as the expression of *potD* was found to be down-regulated in the presence of spermidine but up-regulated in the presence of putrescine in a low choline medium [4, 19, 41].

### Influence of polyamines and PotABCD on the resistance of *S. agalactiae* to acidic pH

The co-transcription of *potABCD* with *clc* and the role of polyamines as key mediators in the resistance to acidic stress of some bacteria could indicate a role of polyamines and of *potABCD* in the acid resistance of *S. agalactiae *. We thus compared the growth of the wild- type strain *S. agalactiae* A909 and the mutant A909∆*potABCD* at the pH of two of its niches (pH 5.5, pH of the intestine; and pH 4.0, pH of the vagina). To this end, these strains were grown in a chemically defined medium supplemented or not with 1 mM spermidine, spermine or putrescine. As polyamines are very basic molecules, these media were buffered at pH 5.5 with 100 mM MES or at pH 4.0 with a 100 mM mix of Na citrate and citric acid to avoid modifications of their pH after the addition of polyamines. Our results revealed no significant difference between the wild-type cells and the mutant at all the tested conditions at pH 5.5 (Fig. S2). As no growth of *S. agalactiae* strains could be obtained at pH 4.0, either in the absence or in the presence of polyamines (results not shown), we compared the ability of the two strains to survive at pH 4.0. To this end, strains A909 and A909∆*potABCD* were incubated in the above cited media and the proportion of surviving bacteria was monitored over time. No significant difference in the survival capacity of the mutant A909 Δ*potABCD* in comparison to the wild-type strain was visible, either in the absence or in the presence of polyamines (Fig. S3).

**Fig. 6.**
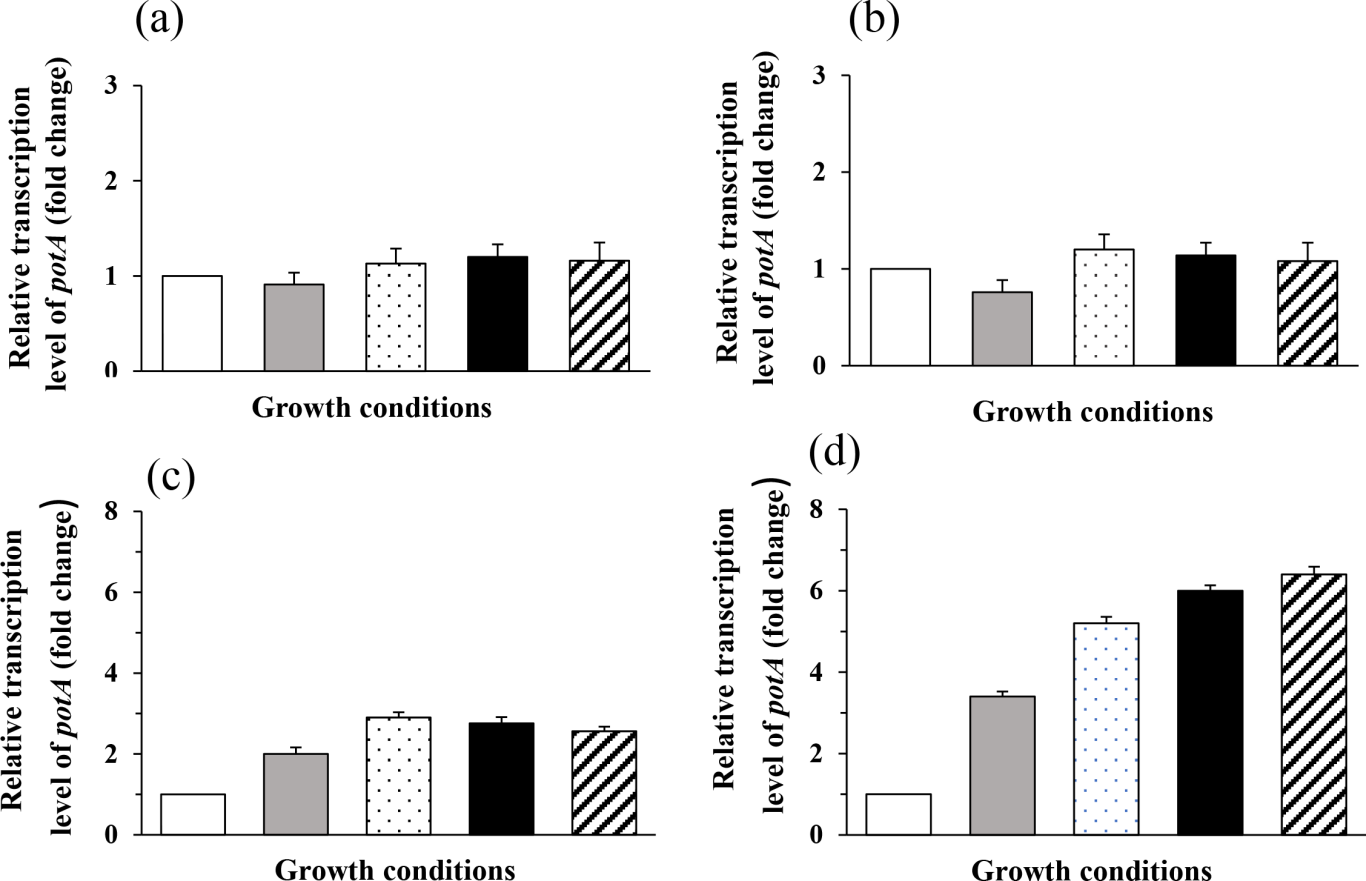
Expression of *potABC*D during acidic and peroxidase-induced oxidative stress in the presence or absence of polyamines. *S. agalactiae* A909 was grown at 37 °C to an OD_600 nm_ of 0.6 (exponential phase) in CDM at pH 7.4. Bacterial cells were harvested (10 ml samples), and then either incubated for 30 min in CDM at pH 7.4 (a and b, white bars), in CDM buffered at pH 5.5 (a; grey, black, spotted and striped bars) and in CDM buffered at pH 4.0 (b; grey, black, spotted and striped bars), or incubated for 20 min (**c**) or 60 min (**d**) in CDM at pH 7.4 in the absence of H_2_O_2_ (white bars), or in the presence of 5 mM H_2_O_2_ (c and d, grey, black, spotted and striped bars). The CDM used for the incubation of the bacteria contained no polyamine (white and grey bars), or contained 1 mM spermidine (dotted bars), 1 mM spermine (black bars) or 1 mM putrescine (striped bars). After incubation, RNAs were extracted and qRT-PCR of *potA* and *recA* transcripts were performed. Transcript levels of each gene were normalized against *recA* transcript levels. Gene expressions are presented as fold change with regard to the level of *potA* transcripts during growth of *S. agalactiae* in normal conditions at pH 7.4 without any addition. Results are presented as means±sd of three independent experiments.

We then compared the expression of the *potABCD* operon at pH 7.4, 5.5 and 4.0 by qRT-PCR. Our results show that acidic stress at pH 5.5 or at pH 4.0 have no effect on the expression of *potA*, either in the absence or in the presence of polyamines (Fig. 6a, b). These expression data correlate with phenotypic observations, since deletion of the PotABCD transporter had no effect on the growth or the survival of *S. agalactiae* under acidic conditions (Figs S2 and S3). However, TLC analyses indicate that both the wild-type strain and the Δ*potABCD* mutant import spermidine and spermine efficiently and putrescine very faintly at pH 5.5 (Fig. S4). Hence, the PotABCD transporter is not involved in the resistance of strain A909 of *S. agalactiae* to acidic stress. However, this property is probably strain-dependent as a microarray analysis of strain 2603 V/R showed that transcription of the *murB–potABCD* operon (but not of the *clc* gene) is increased at pH 5.5 relative to that at pH 7.0 [63]. This induction was found to be dependent of the CsrRS two-component system (CovRS system), which is the major acid response regulator in that organism [64, 65].

### Influence of polyamines and PotABCD on the resistance of *S. agalactiae* to peroxide-induced oxidative stress

In several bacterial species, polyamines, by their function as radical scavengers, were implied in the protection from the toxic effects of reactive oxygen, so we tested if PotABCD has a role in this mechanism in *S. agalactiae* [2–4, 66, 67]. To this end, exponentially growing *potABCD* deletion mutant and wild-type strains were exposed to different concentrations of exogenous H_2_O_2_ (1, 5 and 20 mM). The proportion of surviving bacteria was monitored over time, by plate counts. No significant differences in survival rate were obtained between strain A909 and A909Δ*potABCD* (Fig. S5). We next tested if polyamines are involved in the survival of the same strains submitted to an oxidative stress of 5 mM H_2_O_2_. Again, the wild-type and the mutant strains died at the same rate, whether the growth medium was supplemented or not with spermidine, spermine or putrescine (Fig. S6). We then compared the expression of the *potABCD* operon by qRT-PCR in the absence or in the presence of H_2_O_2_. Hydrogen peroxyde significantly induced the expression of the *potABCD* operon by 2-fold after 20 min of incubation and by 3.4-fold after 60 min of incubation (Fig. 6c). The presence of polyamines in the medium during a peroxidase-induced oxidative stress of 60 min again enhanced the expression of this operon by 5.2-, 6.0- or 6.4-fold in the presence of spermidine, spermine or putrescine, respectively (Fig. 6d).

The above expression data suggest an involvement of PotABCD and polyamines in the resistance of *S. agalactiae* to peroxidase-induced oxidative stress. These data were confirmed by TLC analyses of the intracellular content of cells grown in the presence of polyamines during oxidative stress. In these conditions, the wild-type strain imports spermidine and spermine but also, now, a noticeable amount of putrescine (Fig. 3c). By contrast, only a small quantity of spermine and spermidine are transported by the Δ*potABCD* mutant (Fig. 3d, white arrows). The absence of visible phenotypic effects after deletion of the *potABCD* operon should be explained by this slight transport of polyamines by as yet unidentified polyamine transporter(s). In *S. pneumoniae *, it was also shown that the *potABCD* operon is induced during a peroxidase-induced oxidative stress. The *S. pneumoniae potABCD* deletion mutant had nevertheless a comparable survival rate to the wild-type strain under exposure to the oxidizing stress-inducing agent paraquat [4, 54]. However, different situations exist in the bacterial world as polyamine-deficient mutants of *E. coli* are killed in the presence of concentrations of oxygen that are non-toxic to wild-type cells [3].

### Conclusion

In several bacterial species, PotABCD is able to transport different types of polyamines with different affinities. In a pioneering work on *E. coli,* Kashiwagi concluded that the order of preference of PotABCD is first putrescine, then spermidine and finally spermine [68]. However, this order of preference seems to vary between species [19, 41, 69]. In *S. agalactiae *, depending on the environment of the bacteria, the expression of *potABCD* is induced by different types of polyamines. The import of each of these polyamines thus appears to depend on their affinity towards PotABCD but also on the availability of this transporter, both being governed by the living environment of the bacteria. The suspected redundancy of some polyamine transporters of *Streptococcus* species was suggested to mask some of their phenotypic traits [4, 54]. A similar situation exists also in *S. agalactiae *, making the analysis of these important transporters yet more complex.

## Supplementary Data

Supplementary material 1
