# Supplementary material for: Investigation of the polyamine biosynthetic and transport capability of Streptococcus agalactiae: the non-essential PotABCD transporter
Source: Microbiology (Reading). 2021 Dec 15;167(12):001124. doi: 10.1099/mic.0.001124 (PMC8744998; doi:10.1099/mic.0.001124)
Supplement: Supplementary material 1 [file mic-167-1124-s001.pdf]

Supplementary figures

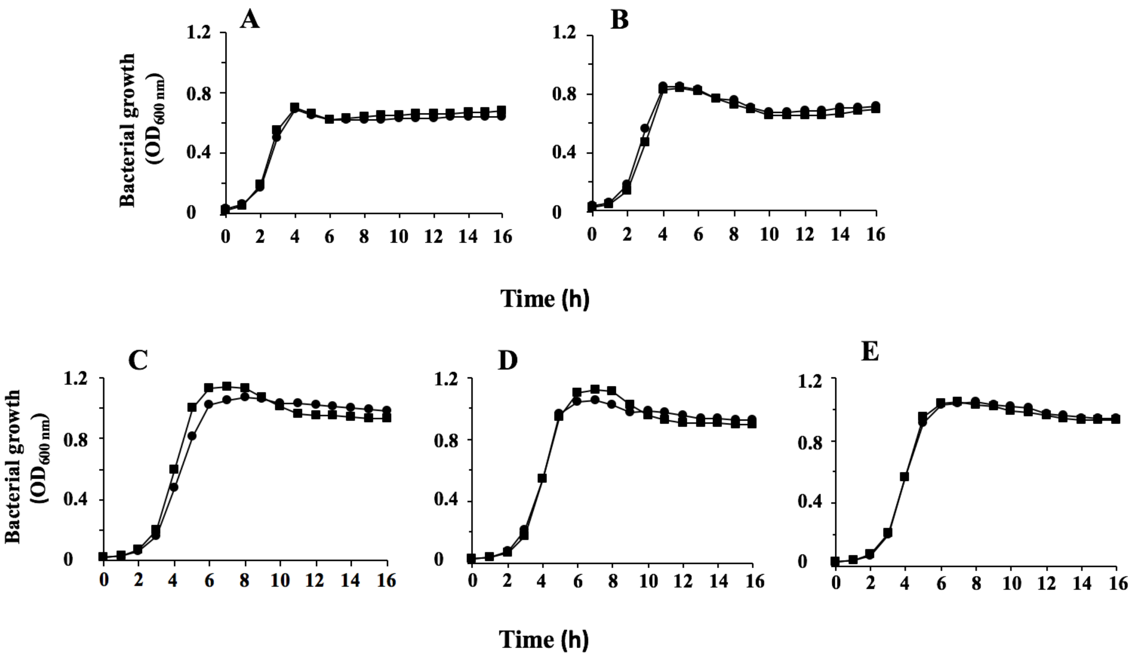

Figure S1. Growth of *Streptococcus agalactiae* strains A909 and A909Δ*potABCD* in a rich or a chemically defined media at pH 7.4. *S. agalactiae* strains A909 (■) and A909Δ*potABCD* (●) were grown at 37°C at pH 7.4 in Todd-Hewitt broth (A) or in a chemically defined medium in the absence of polyamines (B) or in the presence of 1 mM spermidine (C), 1 mM spermine (D), 1 mM putrescine (E). The cultures were incubated for 16 h in microtitre plates (300 µl-culture volume per well) in an Eon thermoregulated spectrophotometer plate reader. The $OD_{600\ nm}$ was measured every hour after double orbital shaking of the plate for 5 s. The reported $OD_{600\ nm}$ is the average OD of three wells inoculated with the same culture. Three independent experiments were made. Standard deviations were always less than 5 %.

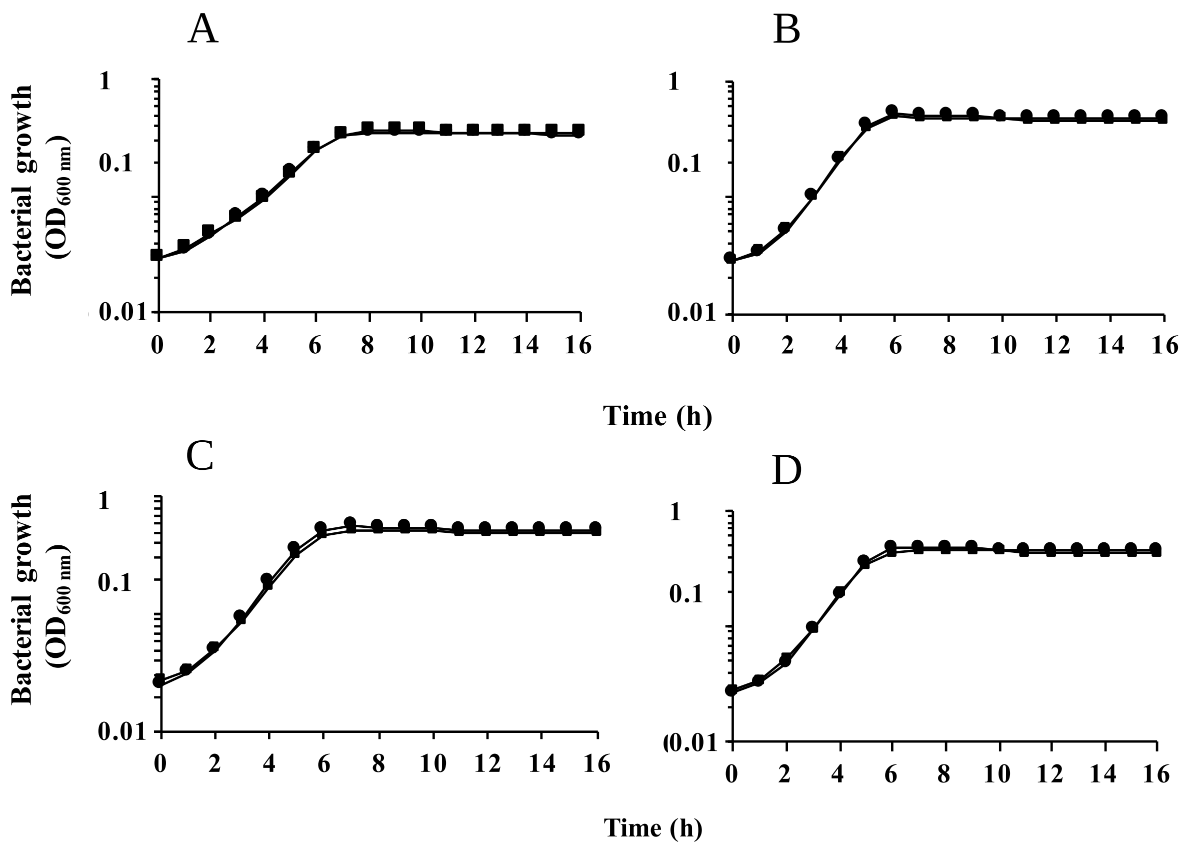

Figure S2. Influence of polyamines on the growth of *Streptococcus agalactiae* strains at pH 5.5. *S. agalactiae* strains A909 (■) and A909Δ*potABCD* (●) were grown overnight in a chemically defined medium without polyamine, washed and used to inoculate a chemically defined medium buffered at pH 5.5 with 100 mM MES and containing either no polyamine (A) or 1 mM spermidine (B), 1 mM spermine (C) or 1 mM putrescine (D). Bacterial cultures were incubated at 37°C for 16 h in microtitre plates (300 µl-culture volume per well) in an Eon thermoregulated spectrophotometer plate reader. The $OD_{600\ nm}$ was measured every hour after double orbital shaking of the plate for 5 s. The reported $OD_{600\ nm}$ is the average OD of three wells inoculated with the same culture. Three independent experiments were realized for all tested conditions. Standard deviations were always less than 8 %.

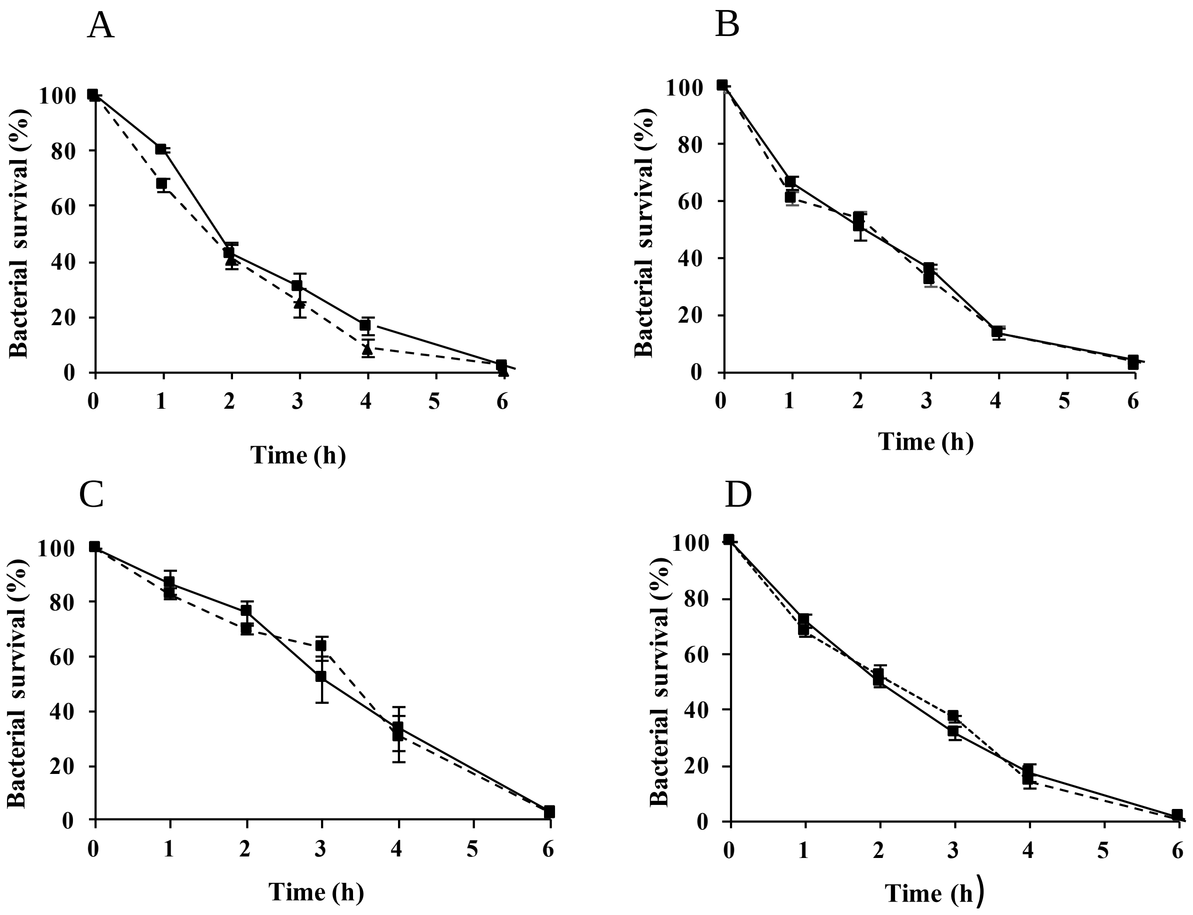

Figure S3. Survival capacity of *Streptococcus agalactiae* A909 and A909Δ*potABCD* at pH 4.0 in the absence or in the presence of polyamines. *S. agalactiae* strains, A909 (dashed lines) and A909Δ*potABCD* (plain lines) were incubated in a chemically defined media buffered at pH 4.0 with a mix of sodium citrate and citric acid and containing either no polyamine (A), 1 mM of putrescine (B), 1 mM spermidine (C) or 1 mM spermine (D). The proportion of each strain was monitored by plating diluted suspensions on TH at suitable time intervals. Results are expressed as percentage of survivors [(number of viable bacteria at the tested condition divided by the number of viable bacteria present in the inoculum multiply by 100]. They are presented as the means ± standard deviations for three independent experiments.

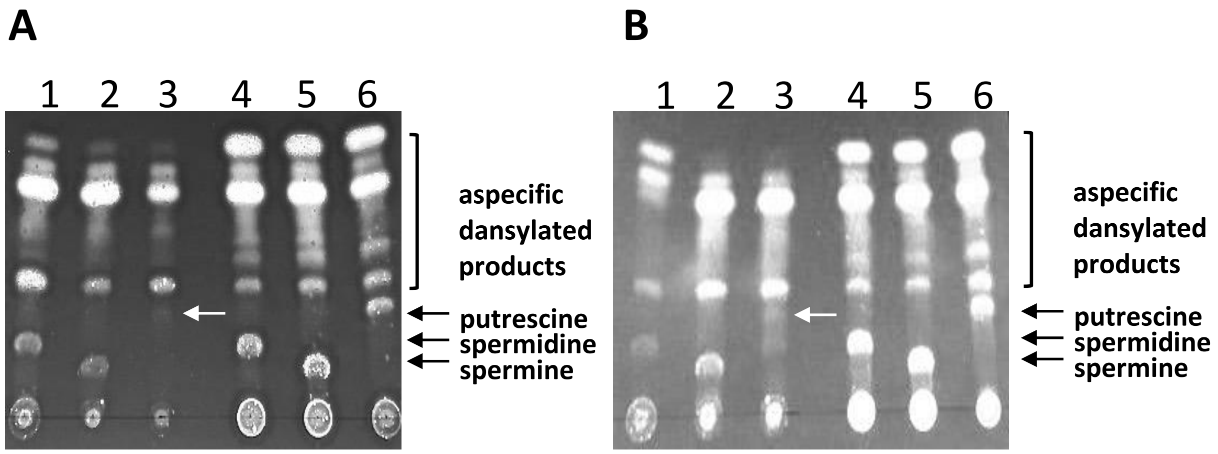

Fig. S4. Intracellular polyamine content of *Streptococcus agalactiae* grown at pH 5.5. *S. agalactiae* A909 (panel A) and A909Δ*potABCD* (panel B) were grown the beginning of the stationary phase in a chemically defined media in the presence of 1 mM spermidine (lanes 1), 1 mM spermine (lanes 2) or 1 mM putrescine (lanes 3). Dansylated bacterial extracts (40 μl) were separated by thin-layer chromatography and photographed under Wood light. Standards of spermidine (0.2 μg), spermine (0.2 μg) and putrescine (0.1 μg) were deposited in lanes 4A and 4B, 5A and 5B, and 6A and 6B, respectively. The white arrows indicate the intracellular presence of a small quantity of putrescine.

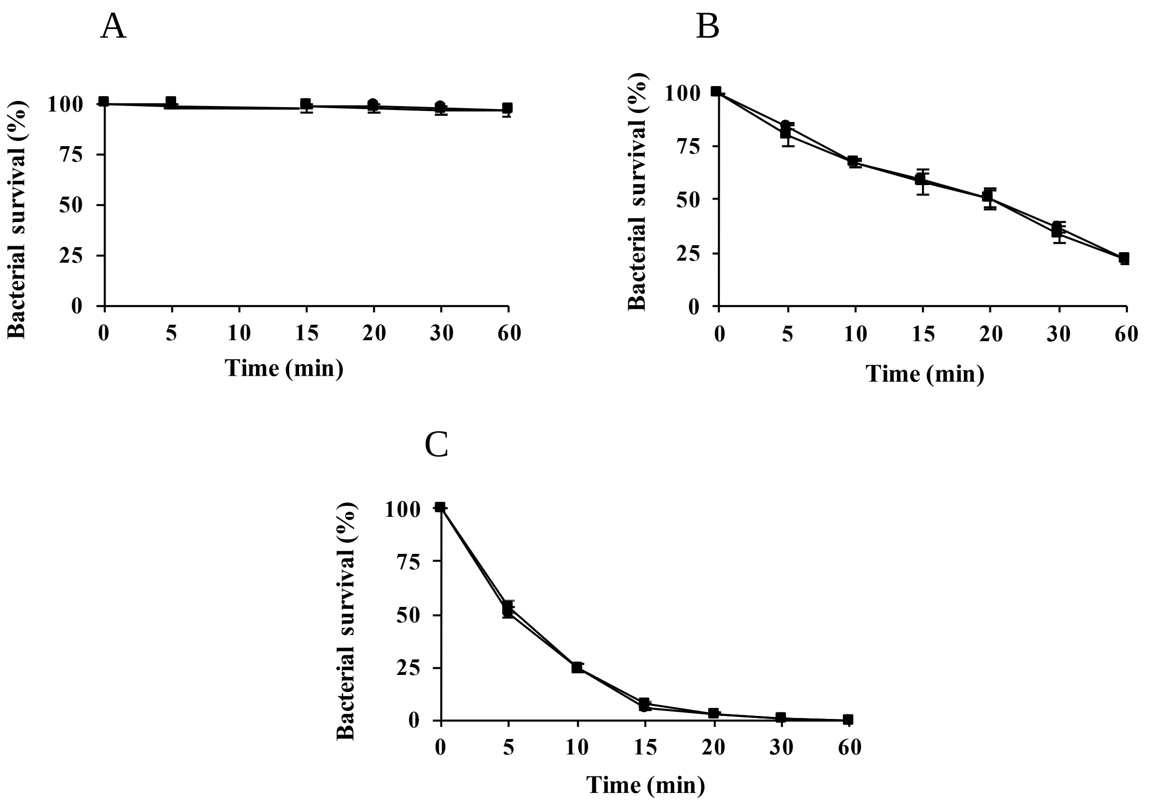

Figure S5. **Survival of strains A909 and A909Δ** ***potABCD*** **during oxidative stress.** Strains A909 and A909Δ*potABCD* cultured in TH broth were submitted to an oxidative stress by the addition of 1 mM (A), 5 mM (B) or 20 mM (C) H_2O_2. The proportion of each strain was monitored during time by plating diluted bacterial suspensions on TH agar. Results are expressed as percentage of survivors [(number of viable bacteria at the tested condition divided by the number of viable bacteria at t0) multiply by 100]. They are presented as the means ± standard deviations for three independent cultures.

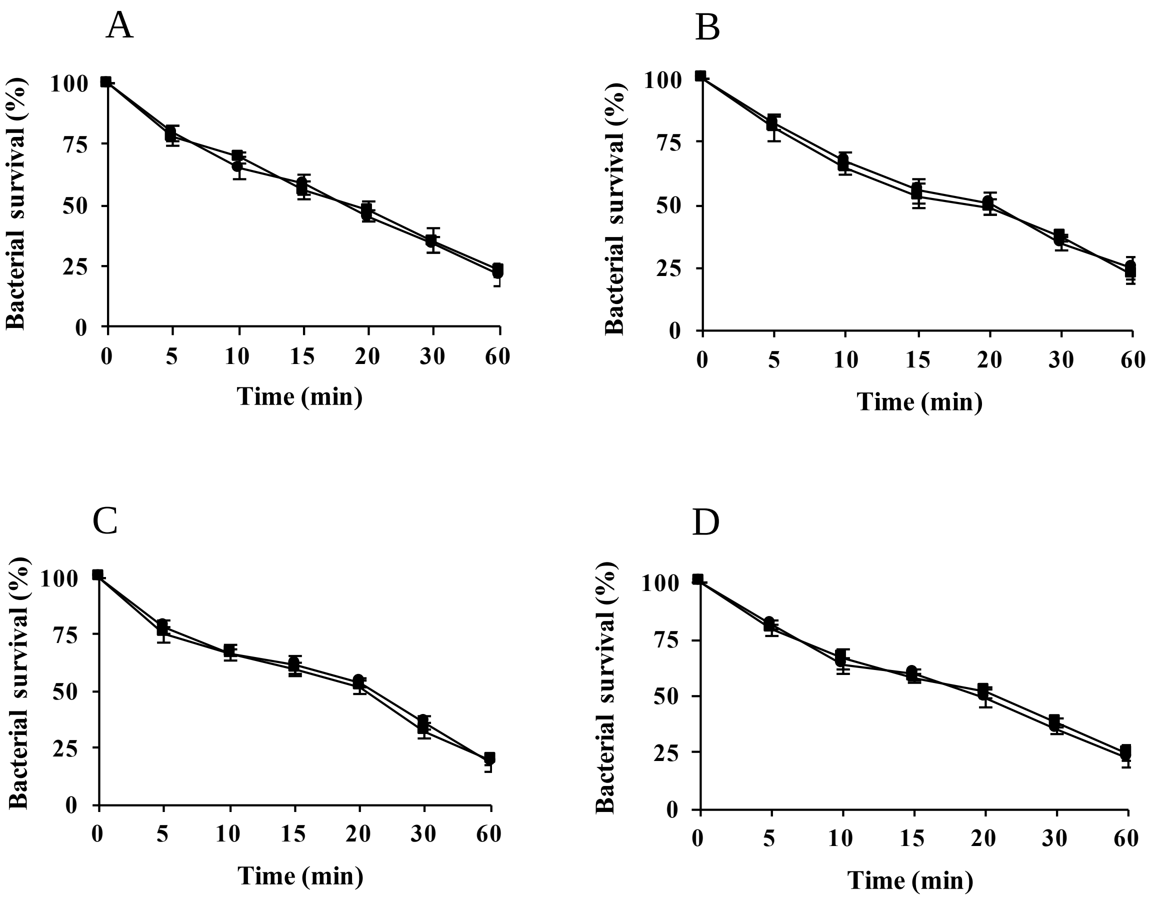

Figure S6. Influence of polyamines on the survival capacity of *Streptococcus agalactiae* strains submited to an oxidative stress. Strains A909 and A909Δ*potABCD* cultured in a chemically defined media in the absence of polyamines were submitted to an oxidative stress by the addition of 5 mM H_2O_2 (A), 5 mM H_2O_2 and 1 mM spermidine (B), 5 mM H_2O_2 and 1 mM putrescine (C), or 5 mM H_2O_2 and 1 mM spermine (D). The proportion of each strain was monitored by plating diluted bacterial suspensions on TH agar at suitable time intervals thereafter. Results are expressed as percentage of survivors [(number of viable bacteria at the tested condition divided by the number of viable bacteria at t0) multiply by 100]. They are presented as the means ± standard deviations for three independent cultures.
